# Multicenter fresh frozen tissue sampling in colorectal cancer: does the quality meet the standards for state of the art biomarker research?

**DOI:** 10.1007/s10561-017-9613-x

**Published:** 2017-03-03

**Authors:** Z. S. Lalmahomed, R. R. J. Coebergh van den Braak, M. H. A. Oomen, S. P. Arshad, P. H. J. Riegman, J. N. M. IJzermans, on behalf of the MATCH study working group, Peter-Paul L. O. Coene, Jan Willem T. Dekker, David D. E. Zimmerman, Geert W. M. Tetteroo, Wouter J. Vles, Wietske W. Vrijland

**Affiliations:** 1 Department of Surgery, Erasmus MC Medical Center, PO Box 2040, 3000 CA Rotterdam, The Netherlands; 2 Department of Pathology, Erasmus MC Medical Center, Rotterdam, The Netherlands

**Keywords:** Colorectal cancer, Biobank, Tissue quality, RNA integrity number

## Abstract

The growing interest in the molecular subclassification of colorectal cancers is increasingly facilitated by large multicenter biobanking initiatives. The quality of tissue sampling is pivotal for successful translational research. This study shows the quality of fresh frozen tissue sampling within a multicenter cohort study for colorectal cancer (CRC) patients. Each of the seven participating hospitals randomly contributed ten tissue samples, which were collected following Standard Operating Procedures (SOP) using established techniques. To indicate if the amount of intact RNA is sufficient for molecular discovery research and prove SOP compliance, the RNA integrity number (RIN) was determined. Samples with a RIN < 6 were measured a second time and when consistently low a third time. The highest RIN was used for further analysis. 91% of the tissue samples had a RIN ≥ 6 (91%). The remaining six samples had a RIN between 5 and 6 (4.5%) or lower than 5 (4.5%). The median overall RIN was 7.3 (range 2.9–9.0). The median RIN of samples in the university hospital homing the biobank was 7.7 and the median RIN for the teaching hospitals was 7.3, ranging from 6.5 to 7.8. No differences were found in the outcome of different hospitals (*p* = 0.39). This study shows that the collection of high quality fresh frozen samples of colorectal cancers is feasible in a multicenter design with complete SOP adherence. Thus, using basic sampling techniques large patient cohorts can be organized for predictive and prognostic (bio)marker research for CRC.

## Introduction

Colorectal cancer (CRC) is the second most common malignancy in the Western World (DeSantis et al. 2014). As in all cancer research, there is a strong trend towards molecular subclassification of CRC (Guinney et al. 2015). The studies conducted to identify these molecular and clinically relevant markers demand large numbers of patients with accurate long-term clinical data combined with high quality tissue samples to be able to use state of the art techniques (Riegman et al. 2007, 2008). Subsequently, the standard enclosed formalin-fixed paraffin-embedded tissue can be used to develop assays for daily clinical practice. Therefore, large multicenter biobanking initiatives are needed to facilitate these research efforts (Burbach et al. 2016; Rose 2016). However, 10% of the fresh frozen tissue samples collected for research purposes are unsuitable for molecular analyses. This is due to multiple non-modifiable factors such as tissue type, intrinsic patient factors, warm ischemia time (extraction of the resection specimen after ligation of the large vessels) and modifiable factors such as cold ischemia time (tissue transport from the operating theatre to the pathology lab), the conservation (fixation/stabilization) method, subsequent transport and the storage of the tissue samples (Boudou-Rouquette et al. 2010; Qualman et al. 2004). The RNA Integrity Number (RIN), first described in 2006, is currently a common standard used to assess tissue quality (Schroeder et al. 2006). This method became well accepted to measure the SOP adherence of quality in tissue banking (Morente et al. 2006).

The current study assessed the tissue quality of the MATCH study, a multicenter cohort study in the region of Rotterdam, the Netherlands, enrolling patients with CRC and obtaining fresh frozen tissue samples in one university hospital with experience in tissue sampling and storage by dedicated personnel, and in six non-university teaching hospitals that are not used to nor standardly equipped and staffed for routine fresh frozen tissue sampling.

## Materials and methods

### MATCH-study design

The MATCH-study is an ongoing multicenter cohort study including adult patients with CRC undergoing curative surgery. The participating centers include one university hospital (Erasmus University Medical Center) and six non-university teaching hospitals (Elisabeth-Tweesteden hospital, IJsselland hospital, Ikazia hospital, Maasstad hospital, Reinier de Graaf Hospital, Franciscus Gasthuis). The MATCH study was approved by the Medical Ethical Board of the Erasmus University Medical Center, Rotterdam, the Netherlands (MEC-2007-088). All patients provide written informed consent for the collection of long-term clinical data and storage of tissue samples. The study is an integrated approach using clinical patient care in non-university hospitals with university-based facilities for tissue and data storage. The rationale of this study was to identify subtypes of colorectal cancer, related prognostic markers and outcome of treatment. Liver metastases was defined as primary outcome defining a good or dismal outcome of disease progression as liver involvement has been demonstrated to be the main factor to determine long term outcome.

### Clinical data

Medical specialists of departments of Surgery, Pathology, Gastroenterology, Radiology and Medical oncology were consulted. Clinical data included reports of colonoscopy, radiology and pathology, as well as surgical reports and postoperative complications. A standard case record was created in a web based multicenter access database. The follow-up of these patients was standardized in all hospitals following an intensive follow-up schedule according the national CRC guidelines (Lochhead et al. 2013).

### Tissue sampling

All tissue samples were handled following a Standard Operation Procedure (SOP) provided by the study team at the start of the study. In short, resection specimens were transported (at room temperature without any conservation fluids) from the operating theatre to the pathology department, immediately following removal of the specimen from the patient. At the pathology department the specimen was handled at room temperature and within two hours after resection samples were snap-frozen as described below. When the 2 h time limit was exceeded, no tissue samples were taken.

Macroscopically, one to four tumor samples and one to two healthy colon tissue samples of 0.5–1 cm^3^ were taken by the pathologist. Tissue sampling for the MATCH study was not allowed to interfere with the standard pathology routine needed for clinical practice. Tumor and normal tissue were stored in labeled cryovials and snap frozen in liquid nitrogen or dry-ice (Mager et al. 2007). Samples were then stored at low-temperature refrigerators (−80 °C) in the hospital of primary surgery and in batches transported to the central tissue bank (−196 °C liquid nitrogen barrels) at the university hospital. Of all new tissue specimens stored in the central bank, on a yearly base 2% is tested for quality, by determining the RNA integrity (Chi et al. 2016; Morente et al. 2006).

### Tissue quality assessment

To assess the tissue quality of the samples collected in the MATCH-study, we randomly selected 10 tissue samples per participating hospital, representing about 4% of the entire collection. Samples that were exposed to neoadjuvant chemotherapy and/or radiotherapy were excluded as this may damage tissue resulting in failure of analysis.

**Fig. 1:**
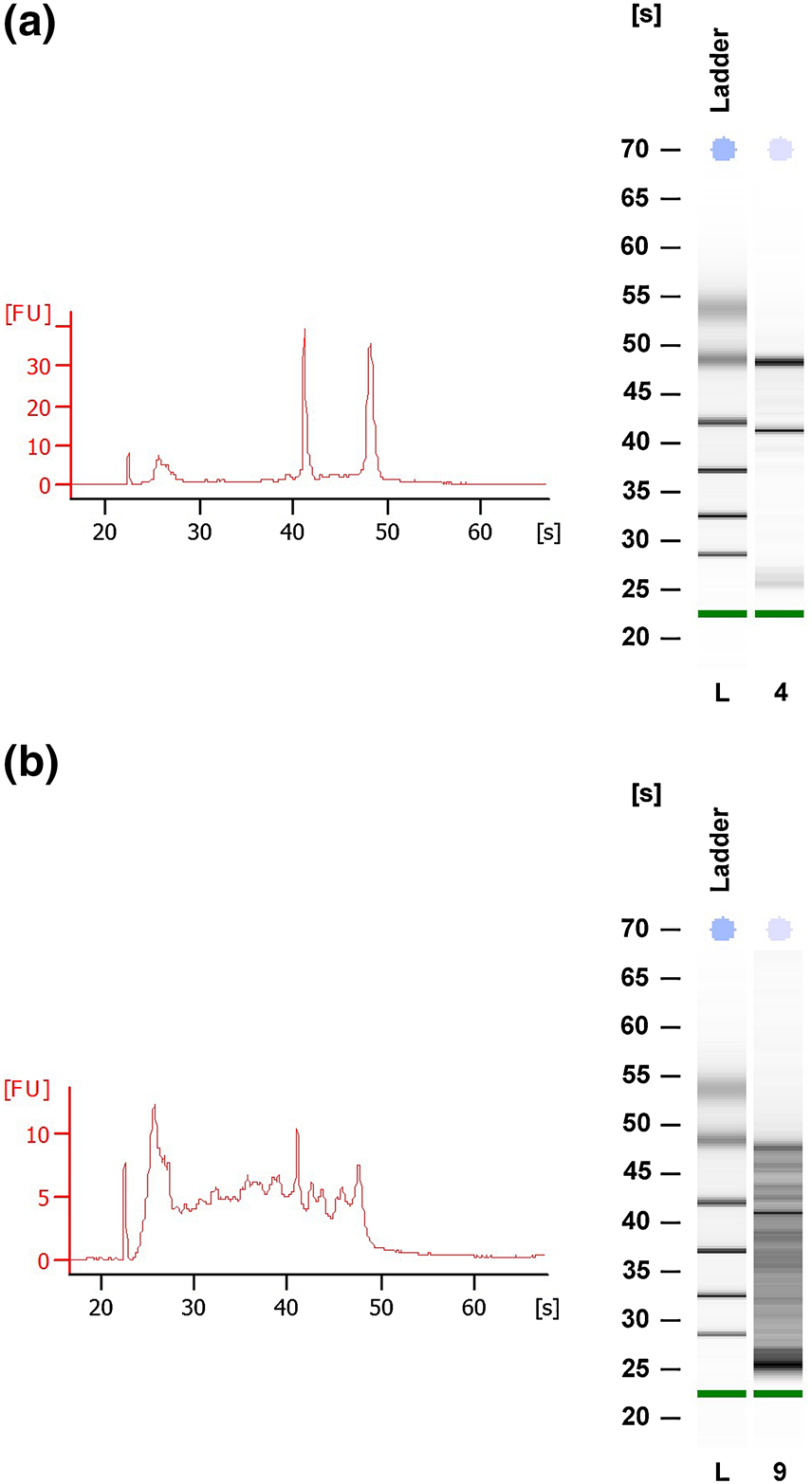
**a** Image intact RNA (RIN 9.0), obtained from the electropherogram and virtual gel. **b** Image partially degraded RNA (RIN 3.3), obtained from the electropherogram and virtual gel

RNA quality was determined by measuring of the RIN (Schisterman et al. 2008; Schroeder et al. 2006). For RNA isolation, 10–20 tissue slides of 10 µm were cut. One slide was colored by hematoxylin and eosin (H&E) stain for morphological confirmation of the diagnosis. For RNA extraction, the slides were put in a Qiazol Lysis buffer and shaken for ten seconds to homogenize the tissue. RNA was then extracted using the miRNeasy Mini Kit (Qiagen, Hilden, Germany) according to the method suggested by the manufacturer. The integrity of RNA was measured by the Bioanalyser (Agilent Technologies, Santa Clara, CA, USA) using the lab-on-a-chip, RNA 6000 nano assay. This is an automated system based on electrophoretic separation. The RIN is directly calculated by applying an algorithm on the ratio of 18S/28S ribosomal RNA bands. A tissue sample with a RIN of ≥ 6 is believed to be of good quality (Fig. 1a) (Strand et al. 2007). Samples with a RIN < 6 (Fig. 1b) were measured a second and if consistently low a third time. When the RIN was still low, the case was discussed with the technician to see if any deviation from protocol (e.g. during the freezing procedure or sample preparation) could explain the low RIN. When samples were measured multiple times, the highest RIN was used for further analysis.

### Statistical analysis

Statistical analyses was performed using SPSS (IBM Corp. Released 2012. IBM SPSS Statistics for Windows, Version 21.0. Armonk, NY: IBM Corp.). Categorical date were described as frequencies with percentages and continuous data as median with the range. The Chi square test was used to compare categorical data, for continuous date the One-way ANOVA test was used. A *p* value less than 0.05 was considered to be statistically significant.

## Results

In total, 70 random samples were selected for analysis out of the 1700 samples collected in the study period 1st October 2007–1st January 2013. During the work-up and data quality check, three samples were excluded leaving a total sample size of n = 67. Two tissue samples were exposed to neoadjuvant radiation therapy and one tissue sample was too small.

**Fig. 2:**
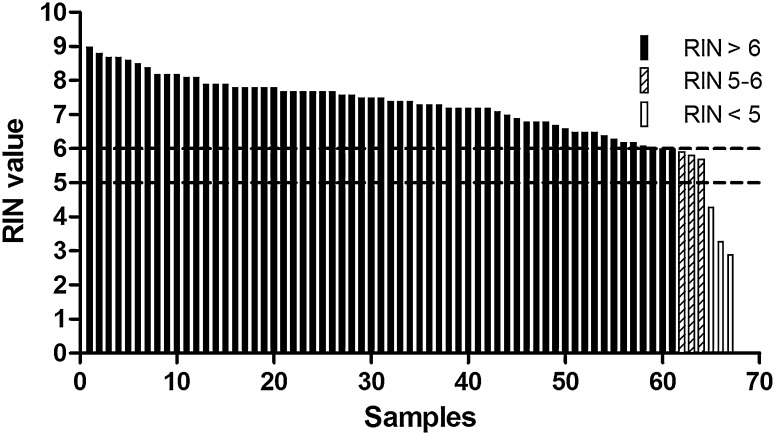
The RIN distribution in 67 samples

**Fig. 3:**
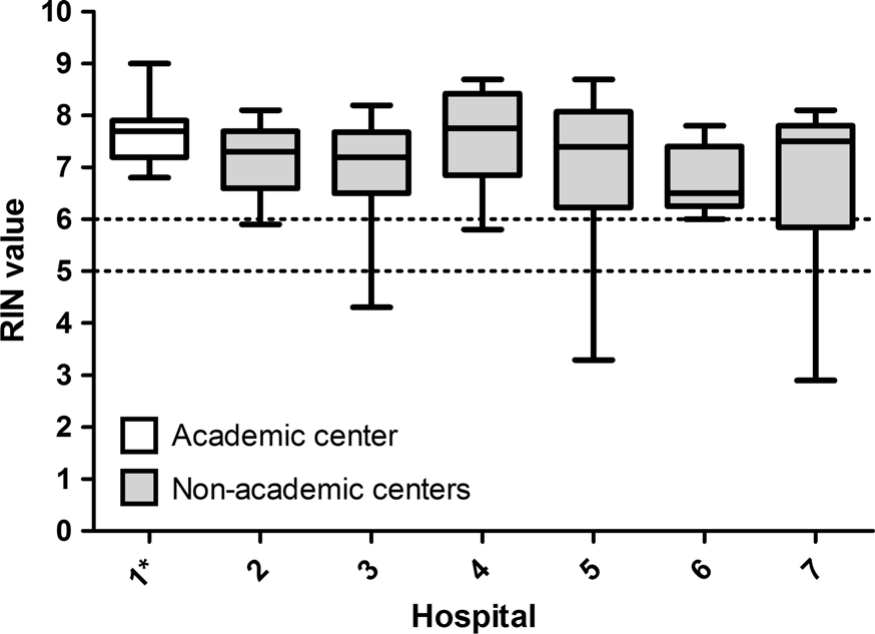
Box plot with the RIN per hospital

**Fig. 4:**
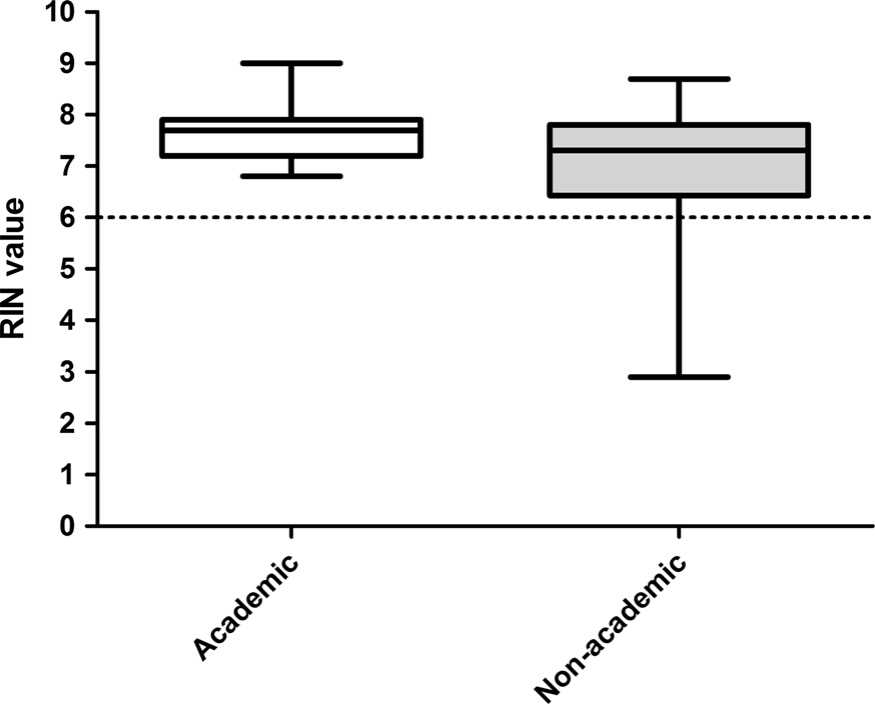
Box plot with the RIN for the university hospital and non-university hospitals

Out of the 67 samples, two samples were analyzed two times (3.0%) and seven samples three times (10.4%). The median overall RIN of all samples was 7.3 (range 2.9–9.0). The majority (n = 61) of the tissue samples had a RIN ≥ 6 (91%). The remaining six samples had a RIN between 5 and 6 (4.5%) or lower than 5 (4.5%) (Figs. 2, 3). Three of the seven samples that were measured three times had a RIN < 5 and were discussed with the technician. However, the low RIN could not be attributed to protocol deviations. The median RIN for a center specialized in tissue sampling (university hospital) was 7.7 and the median RIN for teaching hospitals without a wide experience in this field ranged from 6.5 to 7.8 (Table 1). The overall median RIN of the non-university teaching hospitals (median RIN = 7.3) did not differ significantly with the median RIN of the university hospital (*p* = 0.39) (Fig. 4). When using the specialized university hospital as a reference, the median RIN of one non specialized teaching hospital (hospital 6) had a significantly lower median RIN than the university hospital (*p* = 0.02). However, a median RIN of 6.5 is still well above the cut-off of 6. Interestingly, the range of RIN for the non-university teaching hospitals tended to be larger than the range of RIN if the university hospital (Fig. 3).

**Table 1:** Median RNA integrity number per hospital

Hospital	Number of samples	Median RIN	Range	*p* value
1: University hospital	10	7.7	6.8–9	0.391
2	9	7.3	5.9–8.1	
3	10	7.2	4.3–8.2	
4	10	7.8	5.8–8.7	
5	10	7.4	3.3–8.7	
6	9	6.5	6–7.8	
7	9	7.5	2.9–8.1	
All samples	67	7.3	2.9–9

## Discussion

This study shows that the collection of high quality fresh frozen samples of CRC is feasible in a multicenter design including hospitals for which fresh frozen tissue sampling is not part of the daily routine. In our study, 91% had a RIN ≥ 6 and thus can be used for highly demanding gene array assays.

The RIN was developed and published in 2006 to meet the need for a reliable standard to estimate the integrity of RNA samples (Schroeder et al. 2006). A comparison study comparing a subjective evaluation of the electropherogram, the 28S–18S peaks ratio and the RIN showed a superior result for the manual and RIN method over the ratio method (Strand et al. 2007). Nowadays, the RIN is widely used to quantify the RNA quality of samples and select samples for expression analyses. However, the cut-off used to select ‘high quality’ samples varies in literature, ranging from a RIN of 5–7. These cut-offs can be based on the recommendations in a manufacturer manual or on the experience of a lab (Asterand 2006; Bao et al. 2013; Hong et al. 2010; Viana et al. 2013). At our hospital, we use a RIN of ≥6 as the cut-off which qualified 91% of the samples as high quality samples. When samples repeatedly have a RIN < 6, they may be excluded to prevent a transcript specific bias, or analytical or bioinformatics steps specifically dealing with the low quality samples should be included in the methodology (Lauss et al. 2007; Viljoen et al. 2013). Furthermore, samples with a RIN < 6 can still be used for RT-qPCR applications in which only short amplicons are analyzed.

The quality of RNA expression in tissue samples is dependent on multiple factors such as tissue type, intrinsic patient factors, warm and cold ischemia time, the fixation method and the storage of the tissue samples. While tissue type and intrinsic patient factors cannot be modified, other factors (i.e. ischemia time, fixation method and the storage of samples) can be influenced. The RIN can be used to determine large influences during the pre-analytical phase. Smaller differences can be assessed based on RNA expression analyses (Gallego Romero et al. 2014). For fresh frozen samples, the most important factor appears to be the ischemia time and freeze thawing effects after freezing. A recent review specifically addressing the effect of cold ischemia on RNA stability concluded that in most studies only minimal changes in the RIN were observed (≤10%) during a cold ischemia times of 1–6 h (Grizzle et al. 2016). One outlier reported a significantly decreased RIN of 44% in samples with a cold ischemia time of 1.5 h compared to samples with a cold ischemia time of 10 min (Hong et al. 2010). However, the 28S:18S ratios did not significantly differ (Hong et al. 2010). Importantly, the definition of cold ischemia time differed between studies and often the cold ischemia time in the operating theatre was not taken into account. Furthermore, the effects of warm ischemia time are often ignored while they most likely interact with the effects of cold ischemia time. This may be explained by the fact that this factor is hard to reliably score and is considered to be a non-modifiable factor since attempts to minimize warm ischemia time may affect patient care. Such non-modifiable influences can only be documented to obtain a tool for determination of this influence (Riegman et al. 2015). Although we did not specifically assessed the association between ischemia time and the RIN in our study, the maximum cold ischemia time was 2 h since this was included in the SOP. Thus, the high percentage of high quality samples in our study is in line with the current literature. For the few samples with consistently low RIN values, no protocol deviations were found suggesting the low RIN was caused by non-modifiable factors.

Our study shows that SOP compliance was positive in all the cooperating hospitals and high quality fresh frozen tissue sampling is possible in a multicenter setting including both university and non-university hospitals. These findings support the feasibility of emerging large-scale ‘fit-for-purpose’ biobanks to facilitate the increasingly complex field of fundamental and translational cancer research (Burbach et al. 2016; Kap et al. 2014; Rose 2016).

In conclusion, our study shows that the collection of high quality fresh frozen samples of CRC is feasible in a multicenter design and using basic sampling techniques. Thus, large patient cohorts can be organized for predictive and prognostic (bio)marker research for CRC.
